# Supplementary material for: Astrocytes integrate and drive action potential firing in inhibitory subnetworks
Source: Nat Commun. 2018 Oct 18;9:4336. doi: 10.1038/s41467-018-06338-3 (PMC6194108; doi:10.1038/s41467-018-06338-3)
Supplement: Supplementary file 1 — Supplementary Information [file 41467_2018_6338_MOESM1_ESM.pdf]

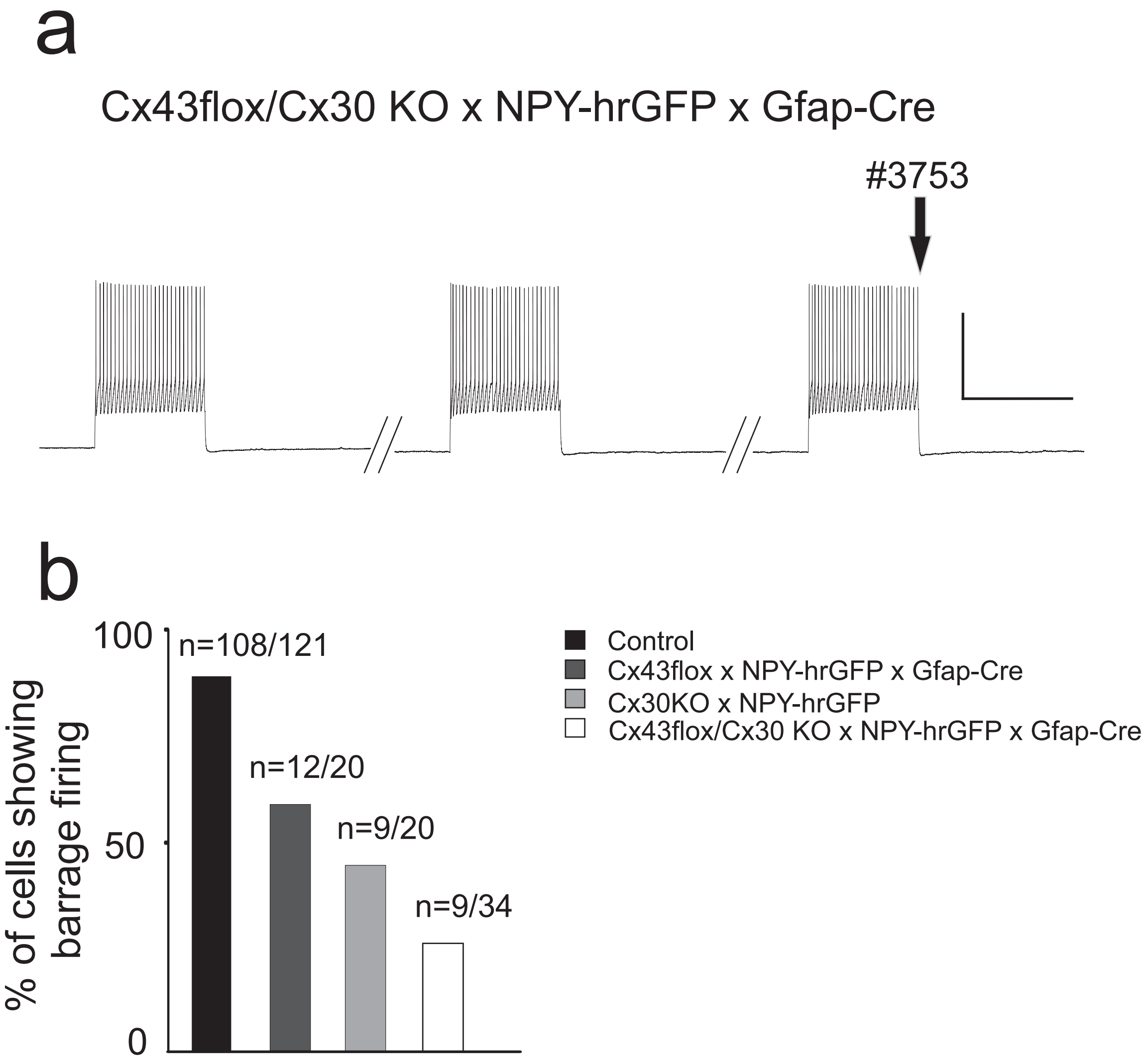

Supplementary Figure 1. Lack of astrocytic gap junction impedes barrage firing induction. (a) Example of response to step depolarization in GFP-labeled interneurons in Cx43flox / Cx30 KO xNPY-hrGFP x Gfap-Cre line. Scale bars, 40mV, 1s (b) Comparison of the percentage of barrage firing in 4 different mice lines.

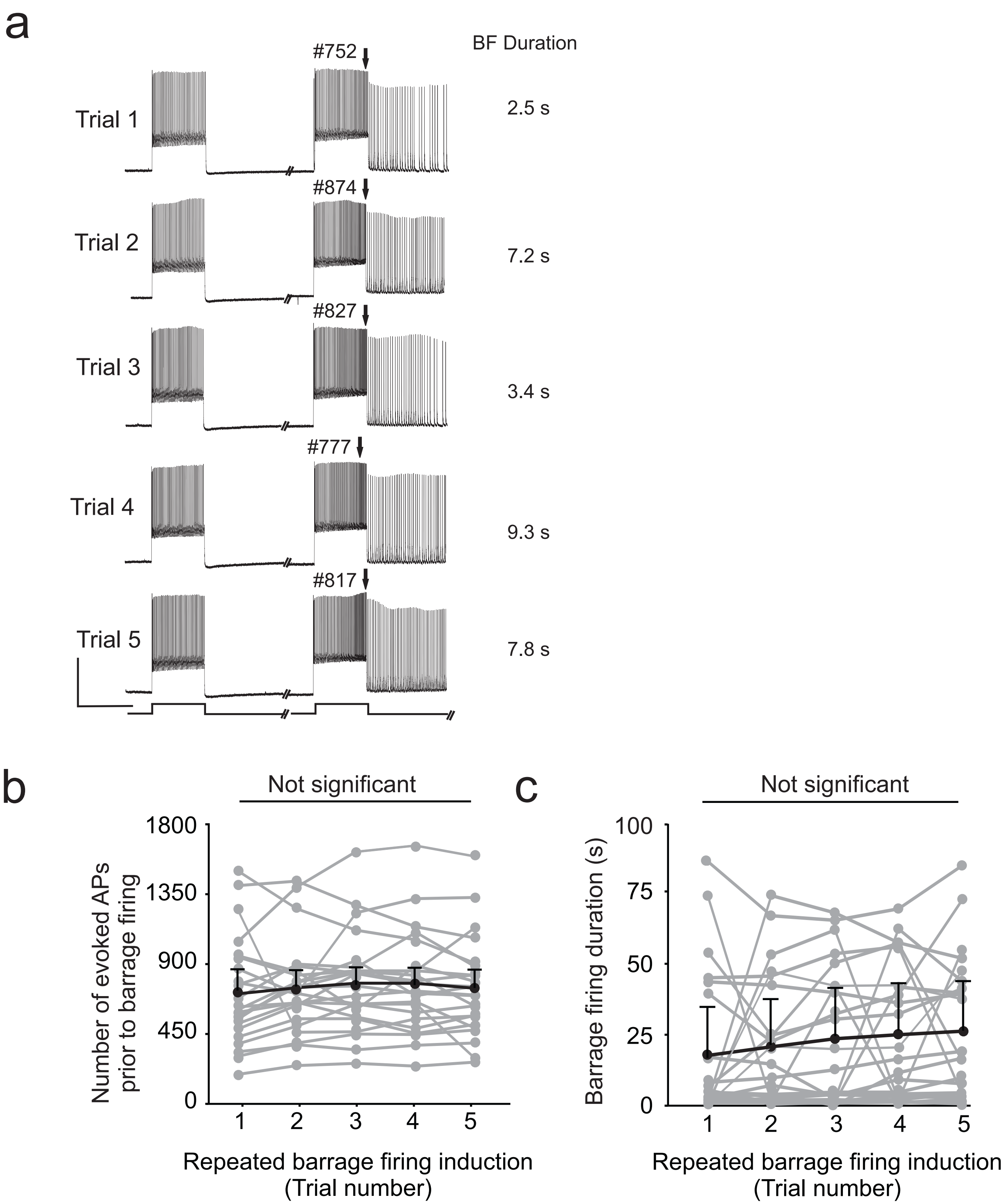

Supplementary Figure 2. Repeated induction of barrage firing in the same NPY interneuron does not affect the number of action potentials required to induce barrage firing. (a) Example of repeated barrage firing induction in an NPY interneuron with five-minute interval between trials. Scale bars, 50mV, 1s (b) The number of evoked spikes to induce barrage firing did not change significantly in consecutive trials ($n = 23$, repeated measures ANOVA; $p=0.53$). (c) There was a trend in increasing duration of barrage firing in the same cell by repeating induction of barrage firing. However, this increase was not significant between trials (repeated measures ANOVA, $p=0.42$).

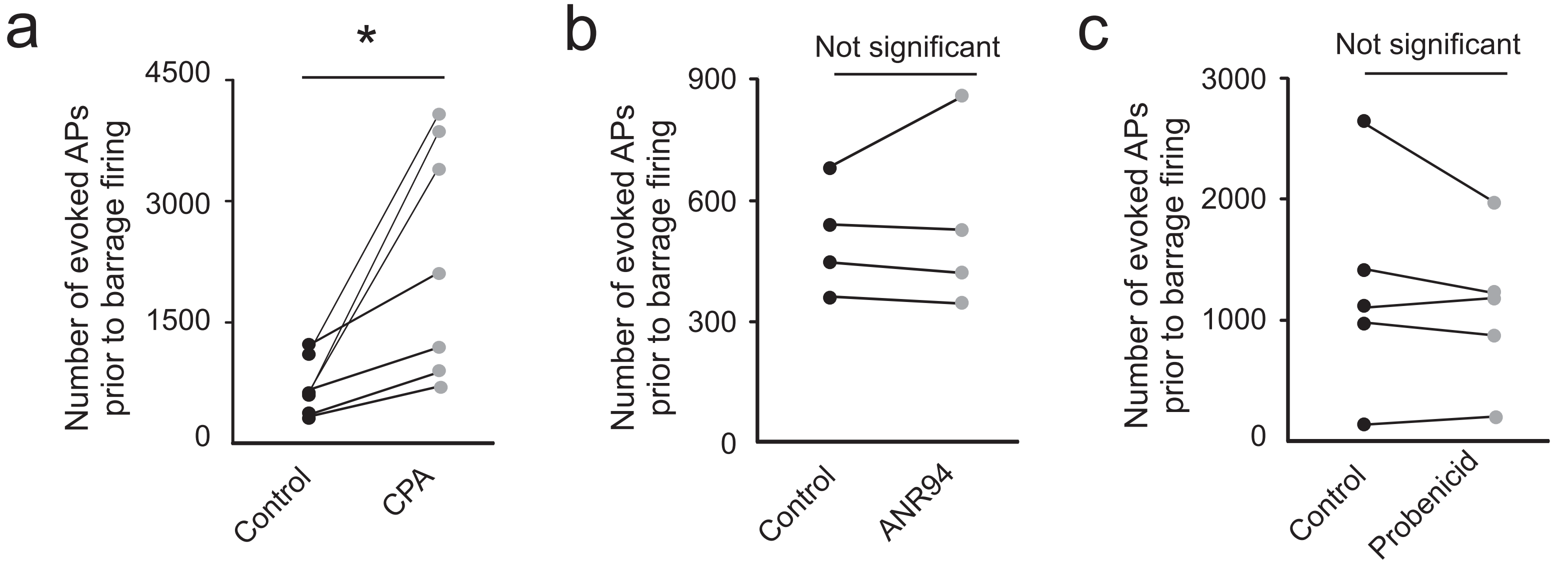

Supplementary Figure 3. Intracellular calcium stores contribute to barrage firing, but adenosine receptors and pannexins do not. (a) Bath application of cyclopiazonic acid (CPA), an inhibitor of the endoplasmic reticulum (ER) Ca2+ATPase, significantly inhibited induction of barrage of firing (n=7; p=0.02). (b) The number of evoked spikes to induce barrage firing did not change significantly following bath application of ANR94 (adenosine/P2 nucleotide receptor antagonist, n=4; paired t test, p=0.6). (c) Bath application of probenecid (pannexin blocker) did not change the number of action potentials required for barrage firing induction (n=5; p=0.3).

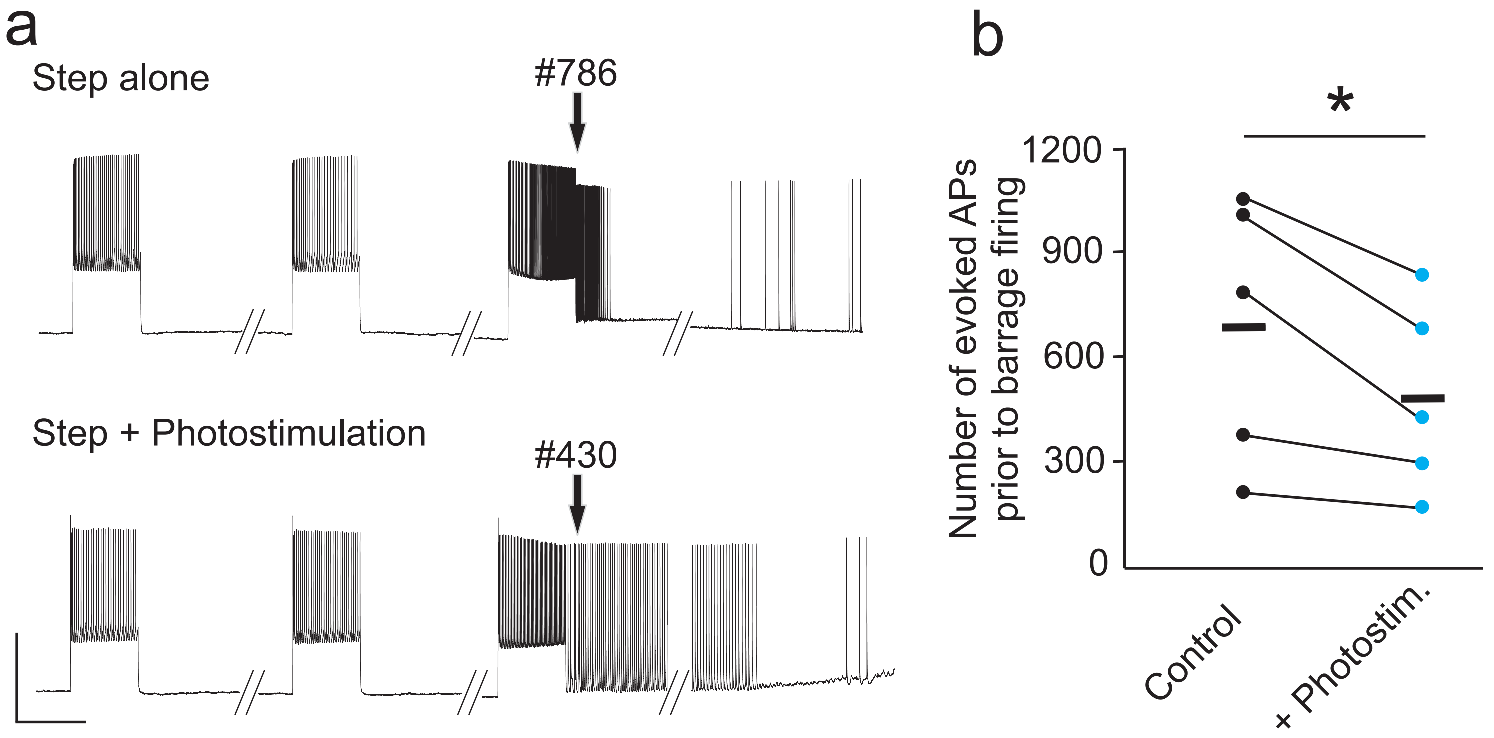

Supplementary Figure 4. Low intensity blue light stimulation facilitates induction of barrage firing
(a) Facilitation of barrage firing generation in an example recording with combined step depolarization and low intensity (2% max power) photostimulation of ChR2-mCherry infected astrocytic network (bottom trace) compared to control condition. The number of spikes required for barrage firing induction is shown on top of the traces. Scale bars, 50mV, 1s (b) Comparison of the number of evoked action potentials (i.e., during step depolarization) required to inducing barrage firing with and without photostimulation (n=5 from 4 mice; *p=0.03).

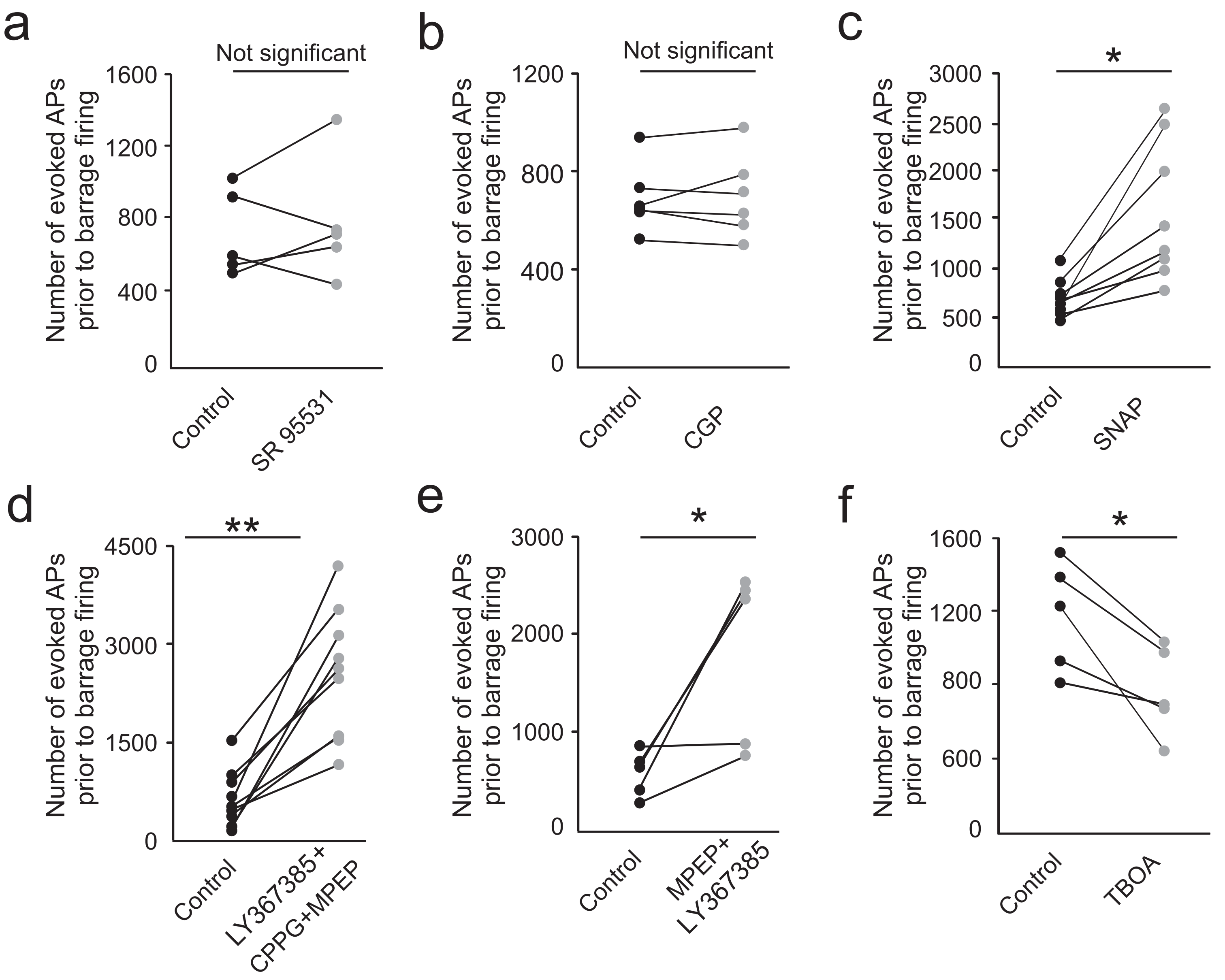

Supplementary Figure 5. GABA and glutamate participate in induction of barrage firing
(a) SR95531(GABAA receptor antagonist) did not affect the number of action potentials required for barrage firing induction (n=5; p=0.6). (b) Bath application of CGP (GABAB receptor antagonist) did not change the number of action potentials required for barrage firing induction (n=6; p=0.8). (c) Bath application of SNAP 5114 to inhibit GABA transport by GAT-3, increased the number of action potentials required for barrage firing induction (n=6; *p=0.02). (d) Bath application of a cocktail of group I/II/III mGluR antagonists completely blocked or inhibited barrage firing induction (n=9; **p=0.003). (e) Bath application of two group I mGluR antagonists was sufficient to inhibit barrage firing induction (n=5; *p=0.03). (f) Blocking glutamate transport by bath application of L-TBOA facilitated induction of barrage firing (n=6; *p=0.02).

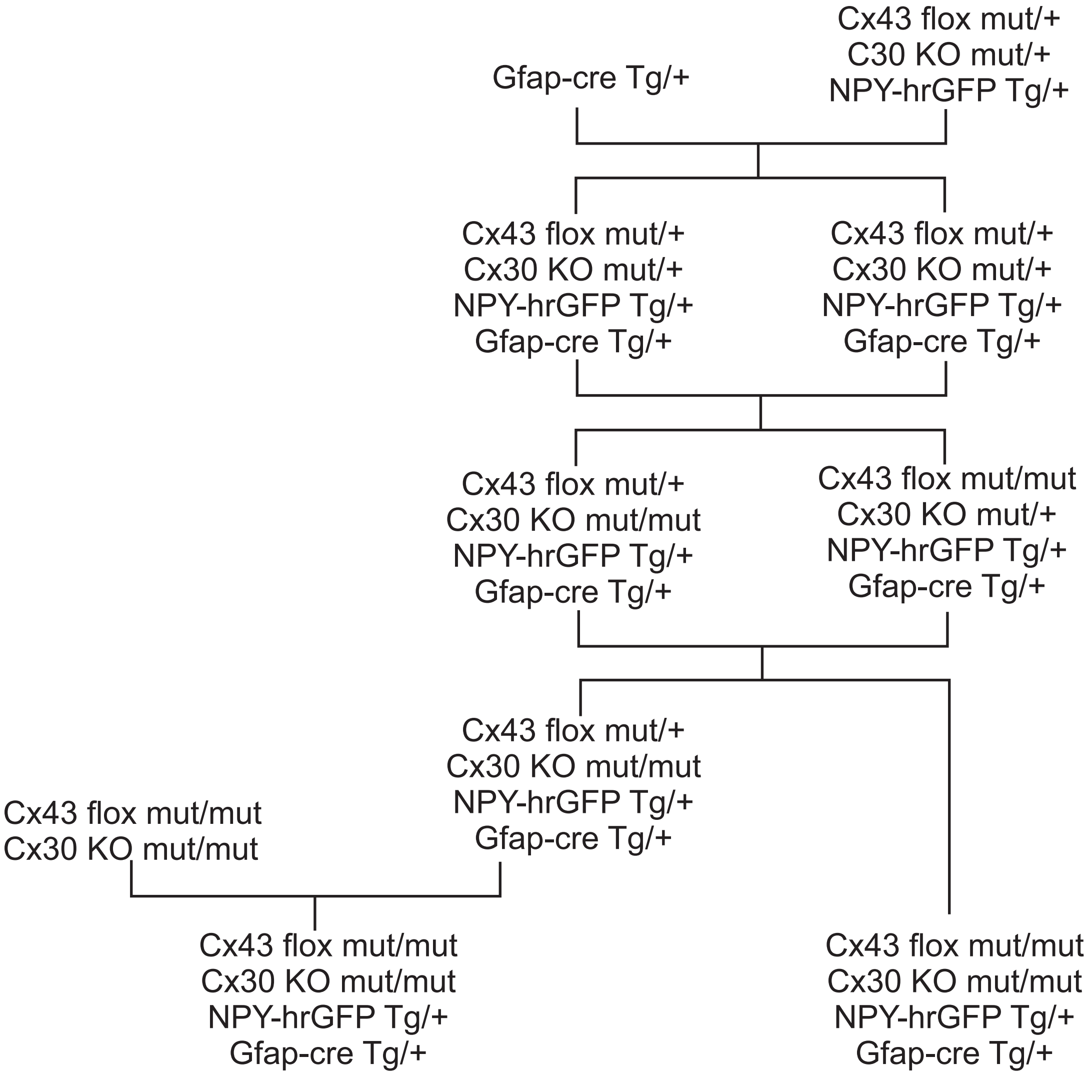

Supplementary Figure 6. Schematic for breeding of double KO mice for Cx30 and Cx43
Simplified schematic showing the steps for generation of mice that expressed GFP in NPY interneurons, were knocked out for Cx30, and were Cx43 KO in astrocytes (Cx43 flox mut/mut; Cx30 KO mut/mut; NPY-hrGFP Tg/+; Gfap-cre Tg/+). Note that animals from different batches were used for mating (not specified in the diagram).

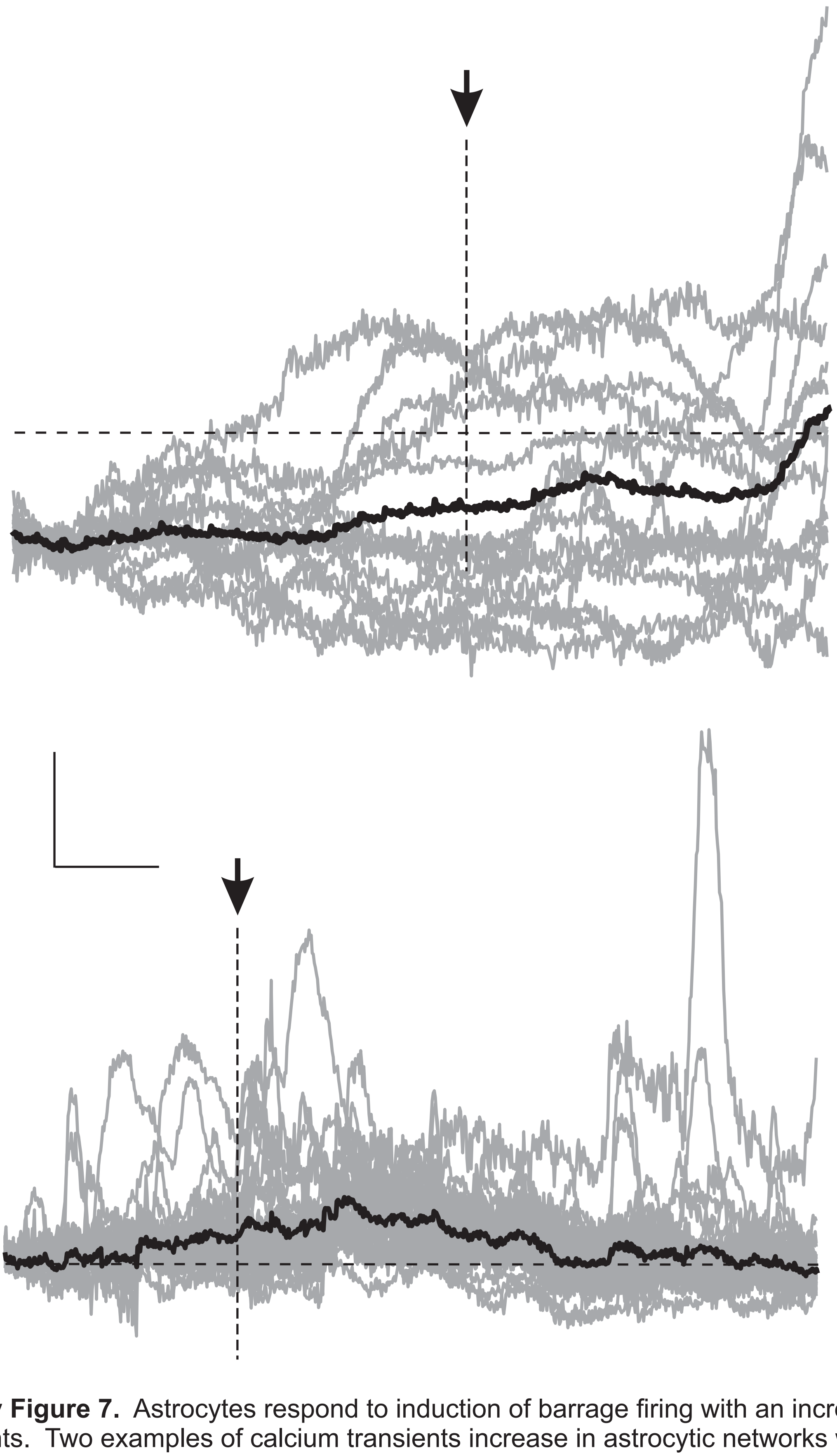

Supplementary Figure 7. Astrocytes respond to induction of barrage firing with an increase in calcium transients. Two examples of calcium transients increase in astrocytic networks with barrage firing induction (2 slices from different mice). The bottom example is the same as in Figure 3c. Each gray trace represents an ROI. Black traces represent average of all ROIs for that astrocyte. Dashed horizontal line represents 0 $\Delta F/F$. Arrows and vertical dashed lines indicate the onset of barrage firing. Scale bars, 5% $\Delta F/F$ and 15 s.
